# Suture of the Tendo-Achillis—A Case*This case was reported and patient exhibited before the late meeting of the Georgia Medical Association, April 19, 1894.

**Published:** 1894-06

**Authors:** Luther B. Grandy

**Affiliations:** Atlanta, Ga.


### SUTURE OF THE TENDO-ACHILLIS—A CASE.*

BY LUTHER B. GRANDY, M.D., ATLANTA, GA.

Accidental division of the tendo-achillis is necessarily of rare occurrence. Tendon surgery, it seems to me, has been somewhat neglected in our text-books and surgical works of reference. Some late journal articles have appeared with very careful directions as to the best method of suturing divided tendons, but some of these methods appear more complicated than ordinary circumstances will require.† Here, as everywhere else, we should aim at simplicity, if by so doing our object and the good of our patient may be accomplished as well. The following case will show how simple approximation of the ends of so powerful a tendon as the tendo-achillis may be followed by perfectly satisfactory results, even in spite of unfavorable conditions:

---

*This case was reported and patient exhibited before the late meeting of the Georgia Medical Association, April 19, 1894.

† See lecture by Dr. Augustus Wilson, in *International Clinics*, Volume I., Fourth Series.

August 7, 1893, a young man, carpenter, was working with his adze, astride a piece of timber. In some way, the tool glanced and inflicted a clean-cut wound on the inner and posterior aspect of his left leg, about one inch above the internal malleolus. He was brought at once to my office, and an examination was made, my associate, Dr. B. W. Bizzell, assisting. There was very little hemorrhage. Extension of the ankle was completely lost. For about one inch and a half above the incised wound, there was a deep depression, which corresponded with the gap between the ends of the divided tendon. A complete division of the tendo-achillis was, therefore, easily detected. We decided upon immediate suture. The patient was etherized and a longitudinal incision made long enough to enable me to reach and bring down the retracted upper portion of the tendon. A stout braided silk ligature was then passed though the two ends of the tendon, about three-fourths of an inch from the cut surface, and on either side of this ligature a stout catgut ligature was passed. Thus the ends were evenly, but not tightly approximated. The external wound was then closed with interrupted silk sutures and the proper antiseptic dressings applied. The ankle was put up in plaster of Paris, in a moderate talipes equinus position, a window being left over the site of the wound for subsequent dressings. The skin-wound healed without difficulty. The silk ligature, the ends of which had been left protruding from the wound, came away on the fifth day. At the end of four weeks the patient begged me to remove the plaster, and with some reluctance, I consented to do so, on condition that he would not use his foot for two weeks. When the plaster was removed he could flex and extend the joint fairly well. A certain amount of union had evidently taken place, but this must have been imperfect, as shown later.

Two or three days after the plaster was removed the patient sustained a fall and the ends of the tendon were completely disunited. He again came to my office and presented the same symptoms as before, with the exception, of course, of the external wound. We cut down upon the tendon and again brought the two ends together. This time we used two silkworm gut ligatures, passed anteró-posteriorly and from side to side, the two thus crossing one another at right angles in the substance of the tendon. We concluded to allow these sutures to remain *in situ,* hoping that they would become encysted. The ankle and leg were again put up in the plaster, as before. At the end of six weeks the plaster was removed. The tendons had again united, but two small sinuses were seen, from which some pus was escaping. These sinuses resisted all efforts to make them heal. As the patient lived several miles in the country, I advised him to enter the Grady Hospital so that these sinuses might be thoroughly opened up and packed. He did so, and the necessary operation was done about Christmas, 1893, by Dr. H. P. Cooper, attending surgeon to the hospital. The silkworm gut ligatures were also removed. The sinuses now healed without difficulty. There was some talipes equinus resulting, which, I assured the patient, would gradually disappear.

The gentleman is here for your inspection. There is no talipes remaining, and you can scarcely detect any lameness or other impairment of function. He has returned to his occupation as carpenter, and suffers no inconvenience.

